# Sound abnormally stimulates the vestibular system in canal dehiscence syndrome by generating pathological fluid-mechanical waves

**DOI:** 10.1038/s41598-018-28592-7

**Published:** 2018-07-06

**Authors:** M. M. Iversen, H. Zhu, W. Zhou, C. C. Della Santina, J. P. Carey, R. D. Rabbitt

**Affiliations:** 1 Department of Bioengineering, University of Utah, Salt Lake City, UT USA; 2 Department of Otolaryngology and Communicative Sciences, University of Mississippi Medical Center, Jackson, MS USA; 3 Department of Otolaryngology – Head and Neck Surgery, Johns Hopkins University School of Medicine, Baltimore, MD USA; 4 Biomedical Engineering, Johns Hopkins University School of Medicine, Baltimore, MD USA; 5 Department of Otolaryngology, University of Utah, Salt Lake City, UT USA; 6 Neuroscience Program, University of Utah, Salt Lake City, UT USA

## Abstract

Individuals suffering from Tullio phenomena experience dizziness, vertigo, and reflexive eye movements (nystagmus) when exposed to seemingly benign acoustic stimuli. The most common cause is a defect in the bone enclosing the vestibular semicircular canals of the inner ear. Surgical repair often corrects the problem, but the precise mechanisms underlying Tullio phenomenon are not known. In the present work we quantified the phenomenon in an animal model of the condition by recording fluid motion in the semicircular canals and neural activity evoked by auditory-frequency stimulation. Results demonstrate short-latency phase-locked afferent neural responses, slowly developing sustained changes in neural discharge rate, and nonlinear fluid pumping in the affected semicircular canal. Experimental data compare favorably to predictions of a nonlinear computational model. Results identify the biophysical origin of Tullio phenomenon in pathological sound-evoked fluid-mechanical waves in the inner ear. Sound energy entering the inner ear at the oval window excites fluid motion at the location of the defect, giving rise to traveling waves that subsequently excite mechano-electrical transduction in the vestibular sensory organs by vibration and nonlinear fluid pumping.

## Introduction

Babylonian tablets scribed in the second millennium BC describe symptoms of vertigo, nystagmus, nausea and loss of balance – often disabling conditions attributed at the time to demonic possession rather than biology^1,2^. It was not until the Greek Hippocratic Corpus that consideration moved from demons to derangements of normal physiology to explain neuropsychiatric phenomena, clearing the path to establish a scientific understanding of balance disorders. The first key discoveries were made in the mid 19^th^ century when Pierre Flourens and Prosper Ménière identified the inner ear semicircular canals (SCC) as the sensory organs responsible for angular motion sensation, and Josef Breuer identified the vestibulo-ocular reflex (VOR) as responsible for compensatory eye movements that stabilize the visual image on the retina by counteracting head movements^3,4^. Normally, SCC afferent neurons exclusively encode and transmit angular head motion information to the brain, but become pathologically sensitive to linear acceleration, vibration, atmospheric pressure, and airborne sound if the temporal bone encasing the vestibular labyrinth is compromised by a fistula or dehiscence. SCC vestibular sensitivity to sound is referred to as Tullio phenomenon, named after Pietro Tullio who discovered that creating a fistula in the bony labyrinth leads to pathological SCC vestibular responses to sound^5^. Patients suffering from Tullio phenomenon experience severe symptoms of sound-induced vertigo and ocular nystagmus. Lloyd Minor and colleagues^6^ identified dehiscence of the superior canal bony labyrinth as the most common cause, which has led to successful methods for diagnosis and surgical repair^7^. But precisely why a fistula or dehiscence of the bony enclosure leads to Tullio phenomenon has remained a mystery for millennia.

Tullio phenomenon is characterized by sound-evoked nystagmus, with the eyes beating primarily in the plane of the affected canal^7–10^. Sound-evoked eye movements are similar to those evoked in normal subjects by continuous angular acceleration of the head, demonstrating that sound evokes tonic semicircular canal responses in these subjects. Recordings from SCC afferent neurons after generating a small fistula in the bony labyrinth have revealed two characteristic types of pathological neural responses to pure tones: (1) neurons that lock action potential timing to a specific phase of the sinusoidal sound wave (phase-locking), and (2) neurons that increase or decrease action potential discharge rate during the sound stimulus without phase-locking (rate encoding)^11^. Phase-locking occurs primarily in neurons that fire action potentials with irregular inter-spike intervals, while rate encoding primarily occurs in neurons that that fire action potentials with regular inter-spike intervals^12–14^. The low-frequency VOR relies primarily on inputs from regularly discharging afferent neurons driving the “sustained” vestibular system^15^, while the high-frequency phasic VOR also relies on irregular phase-locking afferent neural inputs driving the “transient” system^13,16^. Understanding how sound evokes inappropriate sustained and phase-locked vestibular inputs to the brain is therefore essential to understanding eye movements and origins of Tullio phenomenon.

A fistula or dehiscence is thought to give rise to Tullio phenomenon by introducing a flexible window in the bony labyrinth that diverts sound energy away from the cochlea and toward the affected canal^5,17^. The temporal bone encasing the inner ear normally has only two flexible windows, both located in the middle ear. The oval window transmits sound from the middle ear stapes to the cochlea, while the round window is the pressure relief point. Introduction of a flexible “third window” diverts acoustic energy away from the cochlea and round window toward the affected semicircular canal, and this energy shunt explains hearing loss for air-conducted sounds caused by the condition^18,19^. The third mobile window also relieves pressure in the perilymph at the point of the fistula, thereby leading to a transmembrane pressure difference between endolymph and perilymph that can deform the membranous labyrinth, producing flow of endolymph that deflects sensory hair bundles in the SCC crista. This mechanism likely contributes to pressure and low-frequency infrasound sensitivity^6,20^, but cannot account for sustained responses of afferent neurons to auditory frequency sound, nor can it account for frequency dependence of the magnitude and direction of eye movements. It has been suggested in previous studies of Tullio phenomena that nonlinear wave-driven fluid streaming^21^ or Liebau impedance pumping^22–24^ might underlie sustained responses to sound. The work of Grieser *et al*.^21^ presents strong theoretical arguments that Tullio phenomena likely has origins in wave mechanics and nonlinear endolymph pumping in the deformable labyrinth. But sound-evoked neural responses measured in animal models and eye movements measured in humans show frequency dependent changes in excitation vs. inhibition that that are not described by current theories.

In the present work we combine theory and experiment to examine the hypothesis that traveling waves in the vestibular labyrinth give rise to phase-locked afferent neural responses by vibrating sensory hair bundles cycle-by-cycle, and give rise to sustained changes in afferent discharge rate by frequency dependent pumping of endolymph. Computational modeling of the human labyrinth^25^ was performed to elucidate biomechanics of the phenomena, and results were used to design specific experiments to confirm biophysics of the phenomena in an animal model. Experiments were performed in the oyster toadfish, *Opsanus tau*, an animal model selected to facilitate *in vivo* recording of afferent neurons and endolymph flow^26^, and to provide reasonable morphological similarity to human^27^.

Experimental data shows steady endolymph pumping in the semicircular canals evoked by auditory frequency stimuli, as well as sustained afferent neuron responses to sound. The direction and magnitude were both frequency dependent, exhibiting multiple peaks and valleys in the auditory frequency spectrum. Results are consistent with mathematical analysis of nonlinear canal biomechanics and explain the origin of both sustained and transient vestibular responses in subjects suffering from Tullio phenomena.

## Results

**Figure 1:**
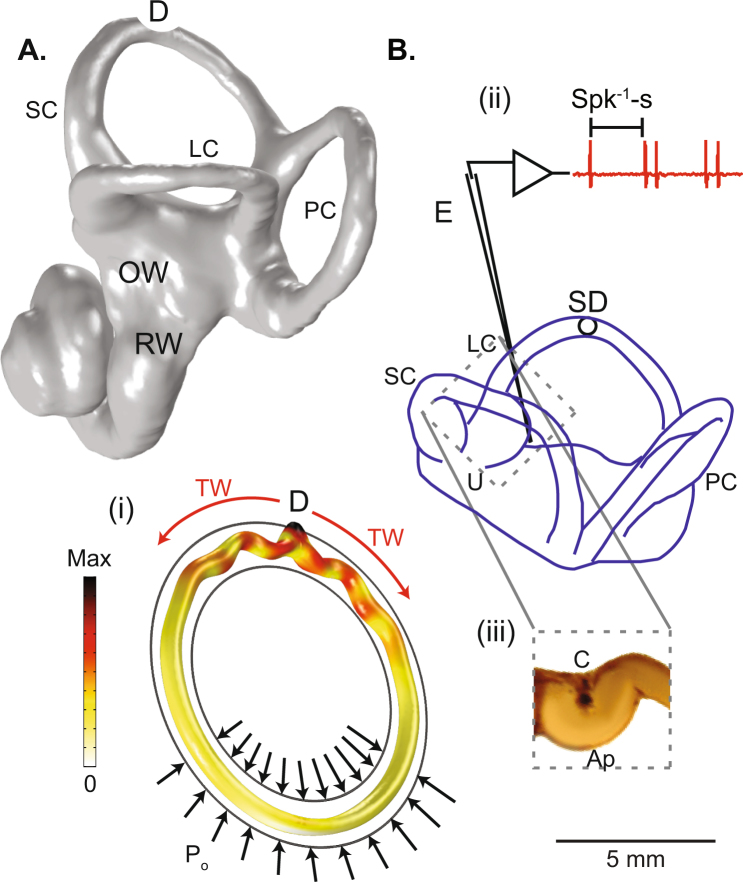
Experimental design. (**A**) Surface reconstruction of an adult human bony labyrinth based on CT images illustrating the oval window (OW) where sound enters the inner ear, round window (RW), lateral canal (LC), posterior canal (PC), superior canal (SC) and location of dehiscence (**D**)^59^. (i) Navier-Stokes simulation with simplified toroidal canal geometry where sinusoidal pressure P_o_ is applied at the outer tube (black arrows) and pressure is relieved at the dehiscence (**D**). The bony labyrinth was modeled as a perilymph-filled rigid tube (black outlines), and the membranous labyrinth was modeled as an endolymph-filled elastic tube. Color bar indicates the displacement magnitude of the membranous duct (white is zero, black is maximum). The maximum displacement occurs at the location of the dehiscence (**D**) and waves propagate away from the dehiscence towards the oval window stimulus site. (**B**) Membranous labyrinth of the experimental animal model (oyster toadfish), showing the location of the simulated dehiscence (SD) in the lateral canal (LC) and location of single-unit afferent neuron recordings in the LC nerve branch (**E**) (Adapted in part from Iversen *et al*.^14^ with the permission of the Acoustical Society of America). (ii) The inverse of the inter-spike-interval, Spk-s^−1^, was recorded. (iii) Close-up of the lateral canal ampulla at the location where velocity fields were measured using PIV.

To gain insight into the potential mechanical origin of Tullio phenomenon we used the finite element method (FEM) to simulate fluid motion and tissue deformation in the vestibular labyrinth. We constructed a simple FEM model based on the geometry of the human superior canal (Fig. 1A, SC) with a dehiscence (D) in the bone located half way around the loop. Endolymph and perilymph were modeled using the nonlinear Navier-Stokes equations and the membranous duct was modeled as a linear elastic tube. The morphology was simplified to an endolymph-filled elastic tube inside a rigid perilymph-filled bony tube (Fig. 1A,i). A pure tone acoustic pressure (1 A i, black arrows) was applied in the perilymph near the oval window (1 A. OW). Simulations predict the presence of traveling waves (TW), propagating away from the location of dehiscence and toward the location of acoustic stimulation P_o_ (See Supplemental Video 1). Waves always traveled away from the dehiscence site and toward the pressure stimulus. This reverse propagation occurs because conservation of mass in the bony labyrinth forces the fluid displacement to be much larger near the small dehiscence relative to fluid displacement in the larger vestibule. Nearly identical results were obtained in our finite element model by applying a prescribed volume displacement at the site of the dehiscence and allowing pressure relief in perilymph at the round window., Hence, because the fluid is essentially incompressible, the mechanics can be examined using a volume velocity stimulus at the round window or an equivalent volume velocity at the dehiscence. This reciprocity motivated our animal model configuration using auditory frequency indentation in the membranous duct to induce endolymph volume velocity at the site of a simulated dehiscence.

**Figure 2:**
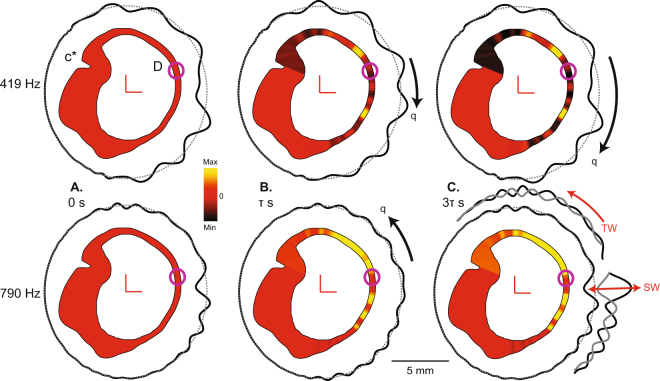
1-D Mechanical simulations. Endolymph pressure and transmembrane pressure in a human lateral canal with simulated dehiscence in response to auditory-frequency stimulation at 419 Hz (top) and 790 Hz (bottom) shown at three instances in time (0, τ, 3τ s, where τ in this simulation was 11.8 s). The spatial distribution of the sustained component of endolymph pressure (Eq. 4, *p*_*e1*_) caused by nonlinear fluid pumping is shown as a color map (black is minimum, yellow is maximum) and the vibrational component of transmembrane pressure (*p*_*0*_ = *p*_*e0*_* − p*_*p0*_) as a polar plot (black solid line relative to gray dotted line). Waves originate from the location of dehiscence and propagate towards the vestibule leading to cycle-by-cycle vibration of sensory hair bundles in the ampulla. Black arrows indicate direction of net fluid displacement, q, predicted to be opposite at 419 vs. 790 Hz. (**A**) At the beginning of the stimulus, waves propagate away from the dehiscence with zero initial pressure gradient across the cupula. (**B**) Over time, waves pump endolymph in a frequency-dependent preferred direction leading to a pressure gradient across the cupula and tonic deflection of sensory hair bundles in the ampulla. (**C**) At 3τ s, the pressure gradient across the cupula reaches maximum while waves continue to propagate away from the dehiscence and vibrate the tissue. At 790 Hz (**C**, lower), transmembrane pressure waves are shown at two instants in time to illustrate traveling waves (TW) on one side of the dehiscence and standing waves (SW) on the other.

Based on FEM simulations we hypothesized that traveling waves give rise to phase-locked afferent responses by vibrating sensory hair bundles as the waves pass through the ampulla, and give rise to sustained afferent responses by sustained pumping of endolymph in the direction of wave propagation. Cycle-by-cycle vibration of hair bundles is clearly supported by the simple FEM model (Fig. 1A,i), but in order for sustained pumping to occur in a preferred direction (e.g. counter clockwise, ampullofugal vs. clockwise, ampullopetal) there must be some asymmetry in the morphology that allows one of the two traveling waves to dominate. To investigate this idea we used the morphologically-descriptive 1-D model of Iversen and Rabbitt^25^ to analyze endolymph pumping. In Fig. 2 the spatial distribution of the sustained component of endolymph pressure caused by nonlinear fluid pumping is shown as a color map and the vibrational component of the transmembrane pressure is displayed in the polar plot. Like the FEM model, waves are predicted by the 1-D model to travel away from the site of simulated dehiscence (Fig. 2) toward the vestibule (See Supplemental Videos 2–3). However, due to the asymmetric geometry, traveling waves on one side of the dehiscence dominate, leading to sustained endolymph pumping in a frequency-dependent preferred direction. The reason why one side can dominate is illustrated in Fig. 2C (790 Hz, lower panel), where the ampullopetal wave reflects as it travels toward the utricle leading to standing waves (SW) while the ampullofugal wave propagates with less reflection. This causes net fluid pumping in the ampullofugal direction. Local reflection of traveling waves is caused by variations in the acoustic-wave impedance introduced by local changes in membranous duct cross-sectional area. The stiffness of the cupula is unimportant relative to fluid mass, fluid viscosity, and membranous duct elasticity for auditory frequency stimuli^25^. Since the wavelength is frequency dependent and the vibrational patterns are frequency dependent^25^, the direction and magnitude of nonlinear endolymph pumping also depend on frequency.

**Figure 3:**
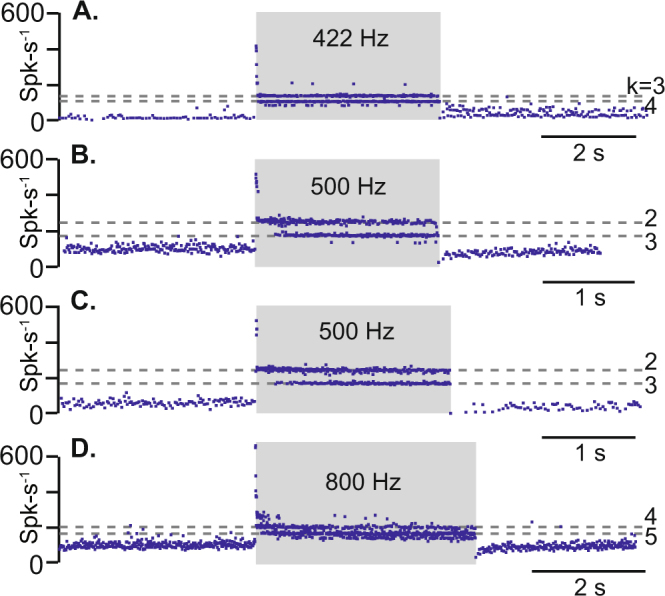
Phase-locked responses of irregularly-discharging afferent neurons in an animal model of canal dehiscence. Irregularly-discharging afferent neurons immediately phase-locked action potential firing at the onset of the auditory-frequency stimulus (average CV for these units was 0.40 +/− 0.19 responding with vector strength 0.85 +/− 0.11). (**A**–**D**) Show responses of individual afferent neurons recorded in the same animal (same animal as Fig. 4). Dotted lines indicate the winding ratio (ratio of action potentials to stimulus cycles j:k) for specified k values. (**A**) Neuron exhibits phase-locking at winding ratios of k = 3, 4 with a 422 Hz stimulus and background firing rate is increased in the tail region after cessation of the auditory stimulus. (**B**,**C**) Two different units that phase-lock to 500 Hz stimulus at winding ratios of k = 2, 3 and exhibit decreased background firing rate in the tail region after cessation of the auditory stimulus. (**D**) Unit phase-locked to 800 Hz stimulus at winding-ratios of k = 4, 5 and also exhibits decreased background firing rate in the tail region after cessation of the auditory stimulus.

If the theoretical predictions in Figs 1 and 2 underlie Tullio phenomena in the living ear, we hypothesized it would be possible to generate both phase-locked afferent responses and frequency-dependent sustained responses in a simple animal model. To test this hypothesis, we recorded single-unit responses of afferent neurons and endolymph fluid velocity evoked by auditory frequency stimuli. The oyster toadfish lateral canal (LC) with a simulated dehiscence (Fig. 1B, SD) was used to facilitate *in vivo* neural recordings and particle imaging velocimetry (PIV)^28^. As expected from simulations, afferent neurons exhibited both phase-locked and sustained responses, depending on the specific neuron and frequency tested. Four examples of phase-locked responses are shown in Fig. 3 for stimuli at 422, 500 and 800 Hz. Notice that these afferents had a rapid onset consistent with the rapid wave propagation from the site of the simulated dehiscence to the ampulla. Phase-locked units also exhibited some adaptation during the stimulus, followed by slow recovery after the stimulus, both consistent with slow endolymph pumping superimposed on the vibration. Consistent with previous reports in this species, SCC afferents that phase-locked to auditory frequency stimuli had irregular baseline inter-spike-intervals, as quantified for this specific population by their high coefficient of variation of 0.40 +/− 0.19^14^. These units phase-locked to the stimulus with high vector strength 0.85 +/− 0.11 and winding ratios between 2–5.

**Figure 4:**
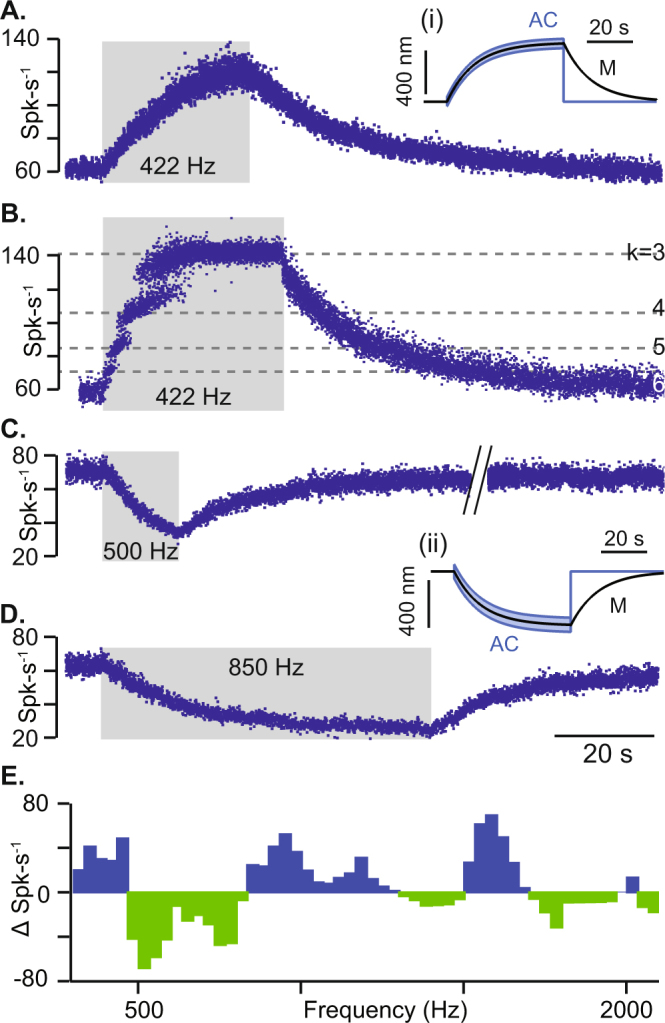
Sustained responses of regularly-discharging afferent neurons in an animal model of canal dehiscence. Regularly discharging afferents responded with sustained increases or decreases in background action potential firing rate that was stimulus frequency-dependent (average CV for these units was 0.05 +/− 0.008 responding with vector strength 0.34 +/− 0.25). (**A**–**E**) show different lateral canal units in the same animal as Fig. 3. (**A**–**B**) Afferents slowly increased firing rate in response to a 422 Hz stimulus and recover after stimulus. (**B**) This afferent also exhibited phase-locking at decreasing winding ratios k = 6,5,4,3 during beginning of stimulus followed by a sustained increase in firing rate. (**C**,**D**) Afferents slowly decreased their firing rate in response to 500 Hz and 850 Hz stimulation,. The average recovery time constant of these units after cessation of the auditory frequency stimulus was 15.04 +/− 1.14 s. (**E**) The change in firing rate of an example regularly-discharging afferent shown as a function of stimulus frequency. (i-ii) Cupula displacement calculated from the 1-D human canal mathematical model where black is the mechanical cupula volume displacement (M) responsible for sustained responses, blue is the cycle-by-cycle cupula vibration at the stimulus frequency (AC) responsible for phase-locked responses.

Responses of regularly discharging afferent neurons did not phase-lock to the auditory frequency stimulus but, instead, showed slow changes in discharge rate consistent with those observed during sustained cupula deflections^26,29^. Four examples are provided in Fig. 4 to stimuli at 422, 500 and 850 Hz (Fig. 4A–D). Notice, like the simulations, sensitivity was either excitatory or inhibitory depending on the stimulus frequency. Afferents that exhibited sustained responses without strong phase-locking had regular baseline inter-spike-intervals with a low average coefficient of variation of 0.05 +/− 0.008. These units responded with low vector strengths of 0.34 +/− 0.25 as expected for regularly discharging afferents^14,30^. One afferent in Fig. 4B responded with mixed-type behavior and phase-locked at decreasing winding ratios during the stimulus onset until reaching a sustained increase in firing rate at k = 3. Responses recovered with approximately the same time constant irrespective of the unit, 15.04 +/− 1.14 s, which reflects the mechanical time constant governing recovery of the cupula to its resting position with very modest neural adaptation in regularly discharging afferents. Whether the response to the auditory frequency stimulus in regularly discharging afferents was excitatory vs. inhibitory was frequency dependent, but not dependent on the specific neuron tested. To further illustrate frequency dependence, the change in firing rate of an example unit is shown in Fig. 4E in response to auditory-frequency stimulation from ~300–3000 Hz. These results are consistent with the hypothesis of frequency-dependent sustained cupula displacement caused by endolymph pumping.

**Figure 5:**
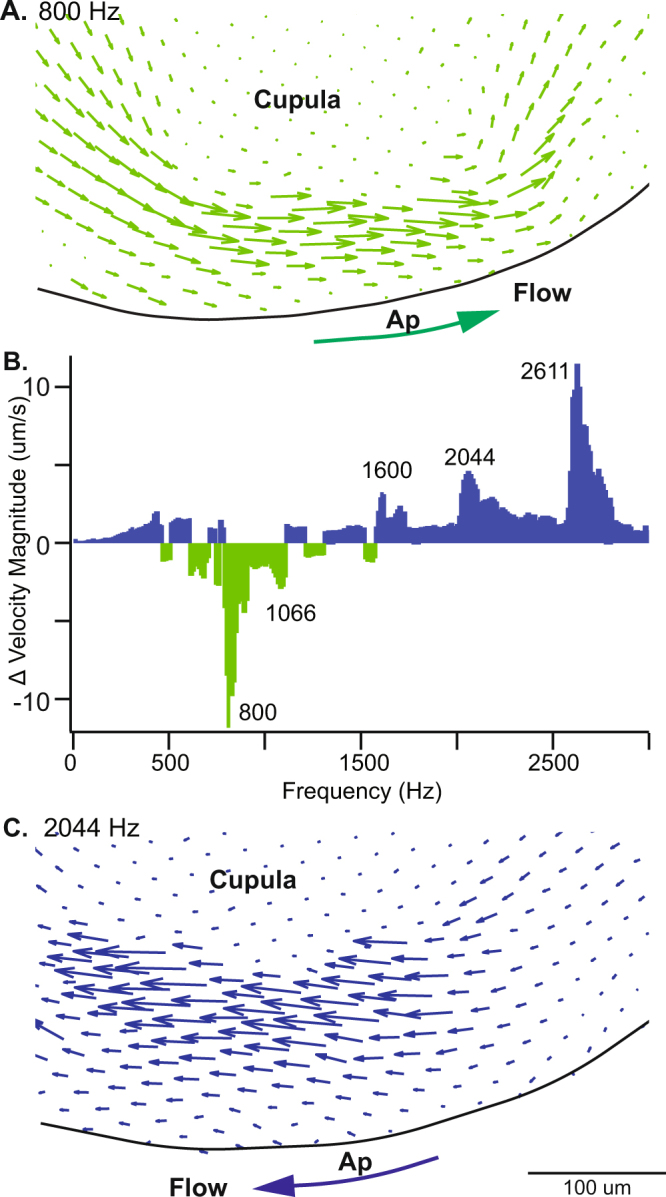
Sustained endolymph pumping in response to auditory frequency stimulation. Particle imaging velocimetry shows sustained endolymph pumping at the apex (Ap) of the lateral canal ampulla flowing over the detached cupula in an animal model of canal dehiscence. (**A**,**C**) Vector fluid velocity fields at the at a peak inhibitory frequency 800 Hz and peak excitatory frequency 2044 Hz show endolymph pumping changes direction. Large arrows indicate direction of net endolymph flow. (**B**) The change in velocity magnitude (µm/s) determined by PIV at the specified frequencies.

To determine if auditory frequency stimuli indeed generate sustained endolymph pumping, we injected fluorescent microspheres into the endolymph through a small fistula in the membranous labyrinth and measured endolymph flow using PIV. For these experiments we dislodged the cupula at the apex of the ampulla (Fig. 1iii Ap) to improve visualization and allow for continuous endolymph pumping around the canal. Dislodging the cupula eliminates the traditional lower corner frequency (~0.06 Hz in the present animal model), but does not change the forces responsible for endolymph pumping by waves in the vicinity of the dehiscence^25^. Supplemental movies show endolymph pumping over the top of the dislodged cupula (Fig. 5 Ap) evoked by auditory frequency stimuli (Supplemental Video 4). Vector fields show steady endolymph velocity in response to 800 and 2044 Hz acoustic stimuli (Fig. 5A,C). Endolymph velocity during stimulation at frequencies from 2 to 3000 Hz shows multiple peak frequencies in excitatory and inhibitory directions (Fig. 5B). Since the frequency spectra are expected to depend on canal morphology, stiffness and stimulation location, as well as on post-mechanical neural signal processing, differences in the spectrum of sensitivity between individual animals and species was expected (e.g. Fig. 4E). Results in Figs 4–5 confirm steady endolymph pumping occurs in a model of canal dehiscence and underlies sustained afferent responses to sound under these conditions.

## Discussion

Present results explain the origin of Tullio phenomena, where a dehiscence or fistula in the bony labyrinth results in pathological and debilitating vestibular responses to sound. Introducing a flexible window in the bony labyrinth alters pressure gradients in the inner ear, which causes hearing loss and introduces abnormal vestibular sensitivity to sound. Conductive hearing loss and vestibular sensitivity to low-frequency stimuli are relatively straightforward to explain on the basis of pressure-driven displacement of inner ear fluids^6,8,9,19^, but sustained responses of SCC afferent neurons to auditory frequency stimuli are more difficult to explain. Here, we present experimental and theoretical evidence that sustained SCC responses to auditory frequency stimuli are due to traveling waves arising at the site of the dehiscenc and propagate in both directions to pump endolymph around the afflicted SCC. Two consequences of these waves combine to generate vestibular sensitivity to sound. First, the waves vibrate the membranous labyrinth, leading to cycle-by-cycle displacement of sensory hair bundles, modulation of mechano-electrical transduction currents, and phase-locked action potentials. Second, traveling waves pump endolymph around the SCC loop^21^, mimicking angular head acceleration, leading to tonic hair bundle displacements, and evoking sustained changes in action potential firing rate. Notably, sound-evoked vibration and endolymph pumping caused by waves are also present in the intact labyrinth, however are decreased by two orders of magnitude or more.

The fact that waves originate at the dehiscence and travel in both directions toward the sound source might seem counterintuitive, but the phenomenon arises directly from the mechanics. Compressibility of the inner ear fluids is negligible at physiologically relevant pressures, so any net inward movement of fluid driven by sound pressure at the oval window must be balanced by an outward movement at the dehiscence and the round window. If the dehiscence size is small compared to the vestibule, the fluid displacement at the dehiscence will be relatively large. Hence, the largest pressure modulation acting across the vestibular membranous labyrinth is at the dehiscence, giving rise to traveling waves propagating away from the dehiscence (e.g. Fig. 1Ai). Because of this, the condition can be modeled experimentally by mechanical stimulation at the site of the dehiscence. Notably, in many cases of canal dehiscence, there is sufficient bone loss to allow dura to herniate into the canal, which defects the membranous labyrinth and induces pulsatile oscillopsia synchronous with heartbeat^31–33^. Frequency-dependent endolymph pumping occurs due to asymmetric traveling wave reflection. Multiple reflections lead to a combination of standing waves and traveling waves in the both limbs of the dehisced canal (e.g. Fig. 2). As a result, pumping occurs ampullofugal or ampullopetal depending on which limb of the canal admits the dominant traveling wave. The direction of wave pumping can switch with frequency (e.g. Figs 4E and 5), because the extent of reflection in the two directions is dependent on tissue morphology and stimulus frequency. Accounting for bi-directional propagation is essential to capture frequency dependence of the direction and magnitude of pumping – features of Tullio phenomena that cannot be explained by unidirectional wave propagation^21^.

The fundamental mechanisms responsible for waves in the vestibular labyrinth are identical to those responsible for traveling waves in the cochlea. In both cases, the system consists of two fluid compartments separated by a flexible partition. The fluids provide the kinetic energy (mass) and the partition provides the potential energy (stiffness), which combine with viscosity to give rise to dispersive traveling waves. In the cochlea, the oval and round windows are positioned in such a way to preferentially excite traveling waves on the flexible cochlear partition, while avoiding excitation of traveling waves on the flexible “vestibular partition”. Introduction of a SCC dehiscence or fistula adds a third flexible window that allows vibration of the oval window to excite traveling waves along the vestibular partition. Since the vestibular system lacks the specialized morphology of the cochlea, there is no equivalent “place principle” or traveling wave frequency decomposition. Nevertheless, there is sound-evoked wave propagation very similar to the cochlea. Waves originate at the dehiscence, propagate in both directions, decay with distance, reflect due to changes in geometry, and combine to generate frequency-dependent patterns of standing and traveling waves. Like the cochlea, cycle-by-cycle vibration of hair bundles leads to afferent nerve responses phase-locked to auditory frequency stimuli. In addition, nonlinear wave pumping evokes slow responses that build up during the acoustic stimulus and decay upon cessation of the stimulus. The same wave pump phenomenon is present in the cochlea, but its effect on cochlear mechanics and potential impact on the sensation of sound has not yet been examined. Avoiding responses to low-frequency wave pumping might be one reason why auditory hair cells evolved to selectively encode to high-frequency signals through the high-pass filter characteristics of their mechano-electrical transduction channels^34,35^. Many SCC hair cells, in contrast, do not have this high-pass characteristic^29^, thus making the canals sensitive to low-frequency physiological stimuli as well as pathological nonlinear wave pumping in dehisced canals.

Experimental evidence reported previously demonstrate that a dehiscence in the superior canal can also increase sound-evoked responses in the horizontal canal and otolith organs^8,11,36–38^. Present results indicate this occurs through the same two mechanisms that affect the dehisced canal – vibration delivered by waves, and wave pumping of endolymph. Waves emanating in both directions from the dehiscence are not restricted to the afflicted canal, but instead are partially transmitted at canal bifurcations to spread throughout the vestibular labyrinth^25,39^. Multiple waves combine to generate complex patterns of sound-evoked standing and traveling waves that vibrate the sensory hair bundles in the sister canals and otolith organs. This increased vibration would be expected to preferentially activate a class of auditory-frequency sensitive otolith afferents with irregular background discharge statistics at rest, thus explaining the decreased thresholds for eliciting vestibular myogenic potentials in patients with a superior canal dehiscence^37,40,41^. In addition to vibration, wave pumping in the afflicted canal stagnates endolymph against the cupula, thus giving rise to a pressure gradient around the canal loop (e.g. Fig. 2). The sound-evoked pressure gradient generated by a superior canal dehiscence, for example, would act at canal bifurcations to evoke tonic endolymph displacement in the horizontal canal. These two mechanical facts likely underlie horizontal canal activation as well as the horizontal component of sound-evoked eye movements in patients with superior canal dehiscence^8,11^.

Results demonstrate why normal physiological function of the semicircular canals and the otolith organs requires the entire vestibular labyrinth to be encased in rigid bone. Any condition that opens an additional mobile window (or windows) in the bony labyrinth would be expected to increase sensitivity of vestibular organs to sound, pressure and vibration because the opening breaks the pressure balance normally present between endolymph and perilymph. Breaking the balance causes pathological responses through deformation/vibration of the membranous labyrinth and nonlinear endolymph pumping. The critical importance of the bony labyrinth likely explains its early appearance as well as uniform presence in early hominids and extant vertebrate species^42–45^. Conditions such as an enlarged vestibular aqueduct or surgically introduced windows are clinical examples that would also break the balance^46^.

It is important to note that the Tullio phenomenon is only one of many symptoms associated with canal dehiscence. For example, patients with a superior canal dehiscence present with an elevated air-bone gap, and increased sensitivity to bone-conducted vibration^9,47^. Present results support two underlying biomechanical mechanisms. First, a dehiscence diverts acoustic power away from the cochlea when sound enters the inner ear via the ossicular chain^19^ and second, a dehiscence increases inner ear fluid vibration in response to bone-conducted vibration. In the intact bony labyrinth, linear acceleration or vibration of the temporal bone generates nearly equal pressure gradients in the perilymph and endolymph that cancel each other and minimize pressure driven deformation of the membranous labyrinth. A dehiscence breaks this balance, and introduces pressure driven vibration of inner ear fluids. For vibration stimuli, the transmembrane pressure is proportional to frequency squared and particularly large at the location of the dehiscence. Based on the present work, this vibration induced pressure imbalance would be predicted to trigger traveling waves and fluid pumping. The traveling wave component would be expected to excite the cochlea via cycle-by-cycle pressure modulation at the oval window and increasing sensitivity of the cochlea to bone conducted vibration.

The mechanisms described here also might explain changes in electrocochleography (ECoG) in cases of superior canal dehiscence, where the short-latency stimulus evoked response (SP) increases relative to the long-latency response (AP)^48^. Sound clicks are known to evoke short latency action potentials in vestibular afferent neurons^49^, and to generate short latency extracellular field potentials. Results support the hypothesis that the ECoG SP response arises in part from high-frequency responses of vestibular otolith organs (increases with dehiscence), while the AP response arises primarily from the cochlea (decreases with dehiscence). Ménière’s disease and other conditions that differentially alter vestibular vs. cochlear responses to sound could also alter the SP/AP ratio.

Our results support the hypothesis that sound-evoked eye movements observed in patients with a superior canal dehiscence arise from both sustained sound-evoked activation of phase-locking irregularly-discharging SCC afferents combined with slowly developing but sustained excitation/inhibition of regularly discharging SCC afferents^11^. The phase-locked afferents would be expected to drive the nonlinear high-frequency vestibulo-ocular reflex (VOR) thus leading to rapid onset slow-phase eye movements^16^. Rapid excitation would almost always be excitatory because vibration-evoked phase-locking is excitatory^11,14,30,50^. The direction of the rapid eye movement would be expected to map primarily to the dehisced SCC with a secondary component arising from the sister horizontal canal. Superimposed on this rapid excitation is a slower component arising from regularly-discharging afferents with onset following the slow time constant of the semicircular canals (e.g. 10–15 s). Present results demonstrate that slow inhibition vs. excitation of these afferents depends on frequency. Sound-evoked neuronal responses are unilateral, so eye movements are further complicated by the inherent excitatory-inhibitory asymmetry of the unilateral VOR. These effects likely combine to explain the relatively fast onset of slow phase eye movements and the predominantly excitatory direction. Upon cessation of the sound, phase-locked afferent responses immediately cease (e.g. Fig. 3) while sustained responses slowly return to baseline following the slow mechanical time constant of the canal (e.g. Fig. 4, tails). Eye movements recorded during this tail period are therefore a more direct measure of sound-evoked cupula displacement, while eye movements during the onset also include a vibration-induced response.

## Methods

### Experimental methods

Oyster toadfish, *Opsanus tau*, (Woods Hole, MA) were prepared using previously published methods^51,52^. The University of Utah Institutional Animal Care and Use Committee approved all animal procedures and all experiments were performed in compliance with relevant policies and regulations. In brief, fish were anesthetized (MS222, 3-aminobenzoic acid ethyl ester; Sigma), immobilized (pancuronium bromide; Sigma), and secured in plastic tank filled with oxygenated seawater. A ~2 cm dorsal craniotomy was made to give access to the vestibular labyrinth and the perilymph was replaced with fluorocarbon (3 M, FC-880). Lateral canal recordings were made with conventional glass microelectrodes. A polished glass pipette fixed to a piezoelectric actuator was used to apply controlled mechanical stimulation of the lateral canal in a location approximate to that of a canal dehiscence (Fig. 1B, SD). Mechanical vibration of the membranous duct at auditory frequencies was used to model sound-evoked responses in a dehiscent canal. This model was motivated by results of our finite-element simulations demonstrating wave propagation away from the dehiscence site as well as reciprocity between stimulation of the dehiscence site vs. the vestibule. Eleven fish contributed useful data.

The regularity of neurons was characterized by the coefficient of variation. Firing rate was defined by the inverse of the inter-spike-interval, Spks^−1^-s, between adjacent action potentials and the mean firing rate ($\mu \ast$) and standard deviation ($\sigma \ast$) of each neuron during spontaneous activity were computed. Coefficient of variation was calculated as the standard deviation over the mean. Phase-locking of neurons was quantified by the winding ratio and vector strength. The winding ratio is defined as the ratio of action potentials fired for each stimulus cycle (j:k). The most sensitive afferent neurons will fire with a winding ratio of 1:1 where an action potential occurs for each stimulus cycle. For example, the neuron in Fig. 3A is responding at winding ratios k = 3 and k = 4. Vector strength was calculated as $r=(1/n)\sqrt{{(\sum \cos {a}_{i})}^{2}+{(\sum \sin {a}_{i})}^{2}}$, where ${a}_{i}$ is the phase angle, and $n$ is the spikes in the interval. Vector strength approaches 0 as the timing of action potential is random relative to the stimulus, and approaches 1 as the timing of the action potential is the same phase for each stimulus cycle.

For particle imaging velocimetry, one micron fluorescent microspheres (Bangs Laboratories, FSDG004) were soaked overnight in lectin (lectin from Triticum vulgaris (Sigma, L9640) and PBS (1 M, Sigma). The microsphere solution was mixed with fluorescent dextran (fluorescein isothiocyanate–dextran; Sigma, FD40S). A small (~20 um) hole was made in the lateral canal ampulla with an electrosurgical generator (Valleylab) set to a 15 W pure cutting waveform with a sharpened tungsten wire as the cutting electrode. The microsphere solution was inserted into the hole with a glass pipette and the hole was then sealed (Locktite, 1710908). Images were collected with an upright microscope (Axioskop Tech, Carl Zeiss, Germany) and a CCD camera (Retiga-EXi, QImaging, Surrey, BC, Canada) at a frame rate of ~150 ms and exposure time of ~100 ms. Images were analyzed in MATLAB 2016a with a time-resolved particle imaging velocimetry tool to calculate endolymph velocity direction and magnitude^53^. Magnitude velocity changes were scaled by the amplitude of the stimulus.

### Modeling methods

We analyzed nonlinear mechanics of the endolymph, perilymph, deformable membranous labyrinth, cupula, and the bony enclosure in the morphology of a single semicircular canal using two complementary approaches: 1) full 3-D finite-element (FE) solution of the nonlinear Navier-Stokes equations coupled to a deformable membranous labyrinth (Comsol, Burlington, MA), and 2) a 1-D wave model of a fluid-filled flexible tube inside a fluid-filled rigid tube^25^. In the finite-element model, we modeled the endolymph and perilymph using the 3-D Navier-Stokes equation including viscosity (*μ* = 8.5 × 10^−6^ kg-s^−1^), density (*ρ* = 1000 kg-m^3^) and the convective nonlinearity. Bulk viscosity was neglected. The membranous labyrinth was modeled as a flexible elastic shell with elastic modulus (*E* = 3 kPa), Poisson ratio (*v* = 0.04) and thickness (*h* = 2 × 10^−5^ m). The bony labyrinth was modeled as rigid with the exception of the fistula, which was modeled using a pressure relief. The model was driven by pressure in the perilymph around the vestibule. The simplified FE morphology shown in Fig. 1 was used. Morphology of the human canal was taken from Curthoys *et al*.^54^. The 1-D model is similar to classical analysis of wave mechanics in deformable blood vessels and fluid filled tubes^55–57^, and is particularly useful because it lends insight into the fundamental mechanism responsible for wave pumping. In the 1-D model, conservation of momentum and conservation of mass applied to the endolymph, perilymph, and the membranous labyrinth results in four coupled partial differential equations for the pressure in the endolymph *p*_*e*_, pressure in the perilymph *p*_*p*_, volume displacement of the endolymph relative to the duct *q*_*e*_, and volume displacement of the perilymph relative to the bone *q*_*p*_, all as functions of time *t* and position *s* along the curved centerline of the toroidal canal. In the present work we were interested in determining how sound at acoustic frequencies leads to steady pumping of endolymph around the toroidal loop. Expanding the 1-D nonlinear conservation of momentum equation for the endolymph (Eq. 4 from Iversen *et al*.^25^) for small changes in cross sectional area provides

1 $${\hat{m}}_{e}(1+\varepsilon \xi ){\partial }_{t}^{2}{q}_{e}+{\hat{c}}_{e}(1+\varepsilon 2\xi ){\partial }_{t}{q}_{e}+{\hat{k}}_{e}{q}_{e}+{\partial }_{s}{p}_{e}=-\,\rho {a}_{se}$$

where ${\hat{m}}_{e}$, ${\hat{c}}_{e}$ and ${\hat{k}}_{e}$ are the effective mass, damping and stiffness parameters, which vary with spatial location along the canal^25^. The right hand side is the forcing arising from angular acceleration of the head, and *p*_*e*_ is the spatially dependent pressure in the endolymph. The nonlinear term arising from the pressure induced change in cross sectional area of the membranous duct is

2 $$\varepsilon \xi =({\partial }_{s}{q}_{e}-g)/{A}_{e0}$$

where *A*_*e*0_ is the undeformed cross-sectional area of the membranous duct and *g(s, t)* arises from mechanical indentation of the membranous duct (which is zero if no indentation is applied). By conservation of mass, ${\partial }_{s}{q}_{e}$ measures the change in cross sectional area (Eq. 1, Iversen *et al*.). This nonlinear term introduces parametrically driven “wave pumping” of endolymph generated by auditory frequency stimuli.

To quantify wave pumping we note that there are two characteristic time scales in the problem: a “fast time” associated with the auditory frequency stimulus, and a “slow time” associated with pumping of endolymph (the equations can be written in nondimensional form and solved using the multi-scale perturbation method^58^ but for simplicity we present an approximate solution here obtained by averaging over the fast time scale). We approximate the endolymph displacement as a sum of two terms, a vibrational term at the fast auditory frequency, and a slower pumping term associated with the characteristic slow time constant:

3 $${q}_{e}={q}_{e0}(s,\omega t)+\varepsilon {q}_{e1}(t)+\mathrm{...}$$

*q*_*e*0_ is the solution of the linear problem when *ε*→0, derived previously in the frequency domain^25^. This is the term that vibrates sensory hair bundles at the auditory frequency and is responsible for phase-locked action potentials. To find the equation for pumping, we substitute Eq. 3 into Eq. 1 to find

4 $${\hat{m}}_{e}{\partial }_{t}^{2}{q}_{e1}+{\hat{c}}_{e}{\partial }_{t}{q}_{e1}+{\hat{k}}_{e}{q}_{e1}+{\partial }_{s}{p}_{e1}=-\,({\partial }_{s}{q}_{e}-g)({\hat{m}}_{e}{\partial }_{t}^{2}{q}_{e0}+2{\hat{c}}_{e}{\partial }_{t}{q}_{e0})/{A}_{e0}$$

We have used the fact that *q*_*e*0_ balances the 0^th^ order pressure gradient and the acceleration forcing. Taking the time average over the period *T* *=* *ω/2π* of the fast time and integrating around the endolymph loop provides the momentum equation for the pumping term

5 $${m}_{e}{\partial }_{t}^{2}{q}_{e1}+{c}_{e}{\partial }_{t}{q}_{e1}+{k}_{e}{q}_{e1}=f$$

The coefficients in Eq. 5 have been integrated around the loop, e.g. ${m}_{e}=\oint {\hat{m}}_{e}ds$. The forcing term on the right hand side arises from auditory frequency stimulus and is given by

6 $$f=\frac{1}{T\varepsilon }{\int }_{0}^{T}\oint \{\,-\,({\partial }_{s}{q}_{e0}-g)({\hat{m}}_{e}{\partial }_{t}^{2}{q}_{e0}+2{\hat{c}}_{e}{\partial }_{t}{q}_{e0})/{A}_{e0}\}dsdt$$

This term is identically zero if the membranous duct is rigid, and hence wave pumping does not occur in rigid fluid-filled tubes. Substituting *q*_*e*0_ into Eq. 6 provides *f*, which is a constant for a given stimulus frequency because of the integrations over both time and space. *f* is nonzero if the membranous duct deforms, but it can have either sign based on frequency and the specific spatio-temporal distribution of membranous duct deformation.

Eq. 5 explains how auditory frequency stimuli generate pumping. Consider the case when an auditory frequency stimulus is suddenly turned on at time *t* = *t*_ON_, and turned off at time $t={t}_{OFF}$. The solution of Eq. 5 in this case is

7 $${q}_{1}(t)=\{\begin{array}{cc}0, & t < {t}_{ON}\\ \frac{f}{{k}_{e}}(1-\frac{{\tau }_{1}{e}^{-(t-{t}_{ON})/{\tau }_{1}}-{\tau }_{2}{e}^{-(t-{t}_{ON})/{\tau }_{2}}}{{\tau }_{1}-{\tau }_{2}}), & t\ge {t}_{ON}\\ \frac{f}{{k}_{e}}(\frac{{\tau }_{1}{e}^{-t(t-{t}_{OFF})/{\tau }_{1}}-{\tau }_{2}{e}^{-(t-{t}_{OFF})/{\tau }_{2}}}{{\tau }_{1}-{\tau }_{2}}), & t\ge {t}_{OFF}\end{array}$$

where the time constants are

8 $$\frac{1}{{\tau }_{1}},\frac{1}{{\tau }_{2}}=\frac{{c}_{e}}{2{m}_{e}}(1\pm \sqrt{1-\frac{4{k}_{e}{m}_{e}}{{c}_{e}^{2}}})$$

In the semicircular canals the slow time constant is about 2 orders of magnitude larger than the fast time constant (*τ*_1_ >> *τ*_2_), so the fast time constant *τ*_2_ ≈ *m*_*e*_/*c*_*e*_, while the slow time constant. *τ*_1_ ≈ *c*_*e*_/*k*_*e*_.

## Electronic supplementary material

Supplemental Video 1

Supplemental Video 2

Supplemental Video 3

Supplemental Video 4
